# Unlocking the Power of Influenza Vaccines for Pediatric Population: A Narrative Review

**DOI:** 10.7759/cureus.55119

**Published:** 2024-02-28

**Authors:** Ahmad Raja S Albalawi, Joud Abdulhamid S Alhassun, Raghad K Almarshud, Hamad A Almejali, Salwa M Alharbi, Amal M Shaybah, Zahra Mohammed A Alshehab, Saleh M Alzahrani, Lama S Abomelha, Alwaleed A Almalki, Abdulrahman O Alkhurayyif, Mariyam S Alalawi, Anwar J Alnass, Khalid F Alzibali, Jehad M Alabdulrahim

**Affiliations:** 1 Family Medicine, King Fahad Specialist Hospital, Tabuk, SAU; 2 Family Medicine, Unaizah College of Medicine and Medical Sciences, Qassim University, Al Qassim, SAU; 3 General Practice, General Administration of Prison Health, Riyadh, SAU; 4 Family Medicine, King Abdulaziz University, Jeddah, SAU; 5 General Practice, Primary Health Care Center, Jazan, SAU; 6 General Practice, Hazem Safwa Primary Health Care Center, Safwa, SAU; 7 General Practice, Al Hazam Primary Health Care Center, Riyadh, SAU; 8 General Practice, Almahalh Primary Health Care Center, Abha, SAU; 9 General Practice, Wadi Alnoman Primary Health Care Center, Makkah, SAU; 10 General Practice, Hotat Bani Tamim Primary Health Care Center, Riyadh, SAU; 11 General Practice, Foudah Primary Health Care Center, Abqaiq, SAU; 12 General Practice, Ibn Sina Primary Health Care Center, Jubail, SAU; 13 Family Medicine, Qassim University, Al Qassim, SAU

**Keywords:** safety, pediatrics, immunogenicity, vaccine, influenza

## Abstract

The flu, often known as influenza, is a dangerous public health hazard for the pediatric population. Immunization is essential for decreasing the burden of the disease and avoiding complications related to influenza. However, the immunogenicity, efficacy, and safety of different influenza vaccines in children warrant careful evaluation.

The purpose of this narrative review is to give a summary of the existing literature on the immunogenicity, efficacy, and safety of several vaccinations against influenza viruses in children. The review incorporates evidence from a range of studies focusing on the outcomes of interest. Immunogenicity studies have shown that influenza vaccines induce a robust immune response in children, primarily through neutralizing antibodies’ formation. However, variations in vaccine composition influence the duration and magnitude of immune responses. Safety is a crucial consideration in pediatric vaccination. In children, influenza vaccinations have generally shown a high safety profile, with mild and temporary side effects being the most common. Vaccinations against influenza have shown a modest level of efficacy in avoiding hospitalizations linked to influenza, laboratory-confirmed influenza infections, and serious consequences in children. Live attenuated vaccines have shown higher effectiveness against matched strains compared to inactivated vaccines. In conclusion, this narrative review highlights that receiving influenza vaccination in children aged six to 47 months is very important. While different vaccines exhibit varying immunogenicity, safety profiles, and effectiveness, they all contribute to reducing the burden of influenza among children. Future research should focus on optimizing vaccine strategies, improving vaccine coverage, and evaluating long-term protection.

## Introduction and background

The influenza virus causes an infectious respiratory illness that infects the throat and nose, and sometimes the virus reaches the lungs. It infects 5% to 15% of the global population annually and causes a serious seasonal respiratory infection [1]. The severity of the disease ranges from mild to severe and may result in death under certain conditions [2]. According to the Centers for Disease Control and Prevention (CDC), the risk of developing potentially dangerous complications related to influenza infection increases among children whose age is less than five years old and children who have chronic diseases [3-5]. Infection rates are high among children; about 20%-30% of the infected population are children every year. This results in an average of 20,000 children younger than five being hospitalized due to flu complications each year [5,6]. One of the most effective methods of preventing the spread of influenza infection and its related complications is vaccination [7,8]. Various influenza vaccines are available, each with its characteristics, including composition, mode of administration, and recommended age groups [9,10].

Influenza vaccines are designed to induce an immune response by stimulating the production of specific antibodies about two weeks after vaccination [9]. Vaccines can protect against the four types of influenza that the research predicts will be most common nowadays [10]. The safety profile of influenza vaccination is generally good in children, but the degree of safety varies according to the type of vaccine, the genetic predisposition, and the target group of the population [11]. Common adverse effects are of grade 1 or 2, such as low-grade fever, aches of the muscles, and soreness at the injection site. These symptoms are temporary and usually subside within two to six days. Serious adverse events from influenza vaccines, such as severe allergic reactions, are extremely rare [12].

Influenza vaccines' effectiveness varies according to several factors, including the match between the vaccine strains and circulating influenza viruses in a given season [13]. Manufacturers predict the most likely strains to circulate annually and formulate the vaccines accordingly. However, due to the inherent variability of the influenza virus, there can be mismatches between the vaccine strains and the circulating strains, affecting the vaccine's effectiveness [14]. Additionally, several other factors can impact the efficacy of influenza vaccines, such as storage conditions, vaccine composition and match, vaccination history, cold chain management, handling and administration, virus variability, adjuvants, and formulation, individual immune response, and timing and seasonality [15]. Proper storage is crucial for maintaining vaccine effectiveness (VE). Vaccines should be stored within specific temperature ranges. Exposure to extreme heat or cold can degrade their potency. Some vaccines are sensitive to light. So, protecting them from direct sunlight is essential [16].

Influenza vaccines' immunogenicity, efficacy, and safety in pediatric populations have been a subject of interest. So, we will review the existing literature to summarize and analyze influenza vaccines' effects on pediatric health.

## Review

Methods

Searches were made on the National Institute of Health PubMed, Scopus, MedLine, and WoS databases between 2012 and 2024, using the phrases (“Flu Vaccine” OR “Influenza Vaccine” OR “Influenzavirus Vaccine” OR “LAIV Vaccine” OR “mRNA-1010” OR “M2e-HBc particle 1818”) AND (Pediatrics OR Neonatology). Articles were screened for their relevance to discussing the immunogenicity, safety, and efficacy of influenza vaccines in the pediatric population at the discretion of the principal researcher. There were no limitations regarding study designs.

Epidemiology of influenza and its impact on children

Influenza viruses have the potential to cause seasonal epidemics of varying intensity each year [17,18]. An estimated one billion instances of seasonal influenza occur each year, with three to five million of those cases resulting in severe disease, according to estimates from the World Health Organization (WHO). Every year, it results in 290,000 to 650,000 respiratory fatalities [17]. Influenza pandemics or worldwide outbreaks are caused only by influenza A and B viruses [18]. Children, especially those aged six months to five years, are considered high-risk for influenza due to their developing immune systems and limited previous exposure to influenza strains [3,17,19,20].

The transmission of influenza among children primarily occurs through respiratory droplets from infected individuals who cough, sneeze, or talk. Children, especially those in close contact environments like schools or daycare centers, can easily spread the virus to others [21,22].

Children who are infected by influenza may experience mild to severe symptoms. Children who have the flu frequently experience sore throat, runny nose, fever, headache, coughing, exhaustion, and occasionally vomiting or diarrhea [23,24]. Generally, children recover from the flu within a week [24]. However, the risk of developing potentially dangerous complications related to influenza infection increases among children whose age is less than five years old, and children who have chronic diseases [3,25]. Complications associated with influenza in pediatric patients include severe breathing problems, secondary bacterial infections like pneumonia, sinus or ear infections, and in some cases may lead to death [23,26]. In rare cases, severe complications like encephalitis (inflammation of the brain), myositis or kidney failure may occur [26]. Influenza infection can worsen pre-existing chronic medical conditions such as asthma or heart disease as does infection with the Mpox virus [27].

Prevention and control strategies for influenza in pediatrics primarily focus on annual flu vaccination [25]. Vaccination is recommended for all children aged six months and older, as it helps reduce the risk of developing flu-related complications and decreases the spread of the virus [5]. Getting vaccinated is not the only preventive action that can help stop the spread of the influenza virus. Other methods include covering the nose and mouth during sneezing and coughing, frequent hand washing, staying at home, and avoiding close contact with ill people [28,29].

Do children without vaccinations have a higher seroprevalence of influenza A and B viruses?

Seroprevalence means the number of members in a population who have specific antibodies against a particular virus. It helps measure the level of immunity to the virus within a population [30]. The seroprevalence of influenza A and B viruses may vary depending on many factors, including age and geographical location which in turn affect population density, community immunity, and the circulating strains of the virus [31].

A cross-sectional study was carried out in the United Arab Emirates to monitor children who have not had a vaccination against the influenza B and A viruses and to give pediatric healthcare workers information that reinforces the community's need for vaccination against influenza [32]. Measurements of IgG antibodies specific to the influenza A and B viruses were conducted between July 2014 and September 2015, involving 294 children with a median age of 4.1 years. Influenza A IgG was reported to be 76.8% negative, 7.4% equivocal, and 15.8% seropositive. The influenza B IgG result was 59.1% negative, 9.6% equivocal, and 31.3% seropositive. This indicates that, across all age categories, influenza B's seropositivity rate was higher than that of influenza A, and that the proportion of children who tested positive for either influenza A or B IgG was 27.9%, and the percentage for both was just 2.7%. The majority of investigated children are serologically naïve and likely to suffer primary influenza illness. Therefore, there is a need for a national policy supporting childhood influenza vaccination [32].

Types of influenza vaccines available for the pediatric population and their composition

Influenza vaccines for pediatrics have been extensively studied, and their compositions vary depending on the specific type of vaccine. Some common types of influenza vaccines used in pediatric populations include the inactivated influenza vaccine (IIV), the live attenuated influenza vaccine (LAIV), Quadrivalent and Trivalent Vaccines, and Egg-Free Vaccines [33].

IIV contains inactivated (killed) flu viruses [34]. It is given to children six months of age or older as an injection [35]. LAIV contains weakened but live flu viruses. It is given to healthy children older than two years who do not have certain medical disorders contraindicating the use of nasal spray (Table 1) [36,37]. Influenza vaccinations are classified according to the number of viral strains they act against [38]. Trivalent vaccinations defend against three strains (one influenza B and two influenza A), whereas quadrivalent vaccines protect against four strains (two influenza A and two influenza B) [39]. Influenza B indicates that Yamagata and Victoria are two immunologically distinct lineages that co-circulate with each other [40]. Since seasons and geographical locations can affect the influenza B virus strain, accordingly, the variation between the vaccine and the widely distributed B lineage is also common [41,42]. QIV, consisting of the two lineages of the influenza B virus, is used to decrease the risk of influenza illness and its associated morbidity and mortality [43] because cross-lineage protection is limited [44,45]. Some children have egg allergies, which can limit their access to certain flu vaccines that are traditionally produced using eggs [46]. So, egg-free versions of influenza vaccines are available and can be considered for children with egg allergies [47]. People with a history of severe egg allergies can still receive influenza vaccines, even if an egg-free vaccine is not available. Everyone aged six months and older with an egg allergy should receive an annual flu vaccine. You can receive any flu vaccine (whether egg-based or non-egg-based) that is otherwise appropriate for your age and health status. No additional safety measures are needed beyond those recommended for any vaccine recipient, regardless of the severity of your previous reaction to the egg. Severe allergic reactions to vaccines are rare but can occur with any vaccine, regardless of allergy history. Ensure vaccines are administered where personnel and equipment for rapid recognition and treatment of allergic reactions are available [46,48].

**Table 1:** Comparing the inactivated influenza vaccine and live attenuated influenza vaccine

Inactivated Influenza Vaccines (IIV) [13,34,35]	Live Attenuated Influenza Vaccines (LAIV) [36,37,49]
IIV vaccines contain killed influenza virus components.	LAIV contains weakened live influenza viruses.
They are recommended for children aged 6 months or older.	LAIV is typically recommended for healthy individuals aged 2-49 years, including children.
IIV is administered via injection.	LAIV is administered via nasal spray.
It has been shown to reduce influenza-related hospitalizations and doctor visits. The vaccine is also effective against outpatient illnesses in children.	Its effectiveness has been found to vary across different seasons and age groups. The vaccine is more effective in children aged 2–6 years in comparison with older children and adults.

Immunogenicity and safety of different types of influenza vaccines in pediatrics

Numerous factors have been found to affect the influenza vaccines' immunogenicity in various populations. Vaccines, adjuvants, individuals, repeated vaccinations, and hereditary variables are some of these factors [15,50]. A Chinese study revealed that age, health status, and history of vaccinations were significant factors influencing the influenza vaccine seroconversion rate in people [15]. Since 2013, the quadrivalent split-virion inactivated influenza vaccination 4 (IIV4) has been available to US residents who are at least six months of age. IIV4 was found to be as immunogenic as TIIV for each of the three common influenza strains and more immunogenic for the additional B strain [51]. A randomized controlled study is being carried out on healthy children aged six to 35 months to administer a half- or full-dose of the IIV4 vaccine [52]. In children between the ages of six and 35 months, it was found that a full dose of IIV4 was as safe and immunogenic as a half dose [52].

The quadrivalent high-dose IIV (IIV4-HD) has been licensed in the USA for use by people 65 years of age and older as of 2019. To examine the safety and immunogenicity of three doses of IIV4-HD with adjuvanted trivalent influenza vaccine (aIIV3) or one to two doses of IIV4 standard dose (IIV4-SD) in children in good health, a phase II clinical trial was carried out [53]. 28-35 days following each dosage, hemagglutination inhibition (HAI) and seroneutralization antibody titers were assessed. With a rise in hemagglutinin dosage, geometric mean HAI titers rose, particularly in children aged six months to three years. When comparing IIV4-SD to IIV4-HD, the GMT was largest for participants who were six months to three years old. These individuals also had the highest seroneutralization antibody GMT ratio. Additionally, unintentional side effects held steady regardless of dosage. There were no documented fatalities or major adverse effects linked to the treatment. Thus, IIV4-HD is more immunogenic in comparison to IIV4-SD and has the same safety profile [54].

An overview of the published systematic review that evaluated the immunogenicity and safety of TIV and QIV against influenza infection was published in 2023 [55]. There were five systematic reviews, with 47,740 individuals in all. The QIV demonstrated superior immunogenicity compared to the TIV in the event of a B-lineage mismatch. QIV was associated with more local side effects in adults, children, and adolescents, even with the equal safety profile of QIV and TIV [55].

An important advancement in the prevention of influenza in young children has been the introduction of adjuvanted and live-attenuated influenza vaccinations [56]. Adjuvants are materials incorporated into vaccinations to boost the vaccination's immunological response [57]. Adjuvant systems 03 (AS03) and emulsions of oil in water MF59 have been used as adjuvants in pandemic monovalent influenza vaccinations and seasonal adjuvanted trivalent influenza vaccines (ATIVs) [56,57]. AS03 is an emulsion of oil and water that includes polysorbate 80, DL-α-tocopherol, and squalene [56,58]. It has been used in the development of pandemic influenza vaccines and enhanced the immune response [56]. Aqua-oil emulsion is another adjuvant that has been utilized in pandemic monovalent and seasonal ATIVs, which is MF59 [56,58]. Squalene, polysorbate 80, and sorbitan trioleate make up its composition [56]. It has been demonstrated that MF59 increases the immunological response to the vaccination and boosts its effectiveness in older people [58]. According to a study that was published in the journal Frontiers in Immunology, children respond well to AS03- and MF59-adjuvanted influenza vaccinations, which also cause strong immune responses [59]. Additionally, the study discovered that these vaccinations work better at generating a longer-lasting antibody response in newborns and young children against both homologous and heterologous influenza strains than non-adjuvanted vaccines [59].

A systematic review and meta-analysis have demonstrated that inactivated influenza vaccinations with MF59 adjuvant are safe, effective, and immunogenic in healthy individuals across a range of age groups [60]. The study examined the effectiveness of non-adjuvanted inactivated influenza vaccines and IIV with MF59 adjuvant against certain influenza vaccine strains in all age categories. According to the study, both in the group of healthy adults and the group of healthy elderly people, MF59-AIV showed superior immunogenicity against specific vaccine virus strains than non-adjuvanted influenza vaccines. Regarding the seroconversion and seroprotection rates of the influenza vaccination, the quality of the evidence is moderate to high [60].

Children aged 6 to 47 months were included in the studies that looked at the immunogenicity and safety of the cell-based QIIV (QIVc). The results showed that QIVc was immunogenic and safe, similar to those of a QIV that was licensed in the US [61]. During the 2019-2020 influenza season in the Northern Hemisphere, an RCT multicenter trial was carried out [61]. Children were assigned to receive either QIVc or QIV and got either one or two doses of the vaccine, depending on their prior history of influenza immunization. Using HAI and microneutralization assays, antibody titers were measured using sera obtained 28 days and 180 days after the prior immunization. Safety was evaluated during this time. Every vaccination strain satisfied the success requirements. In children aged six to 47 months, QIVc was well tolerated, and the immunological responses resembled those of a QIV licensed in the United States. There were no significant adverse events linked to the vaccine, and the rates of adverse events were comparable in the two groups.

According to the CDC, the safety profile of annual influenza vaccines is good for adults and children [62]. Although the CDC supports the safety of influenza vaccines, there are some consequences to considering them. Guillain-Barré syndrome (GBS) within six weeks of a previous dose of influenza vaccine is one of the most important adverse events that should be evaluated with any type of influenza vaccine. Another precaution when receiving an influenza vaccine is the presence of acute disease, either moderate or severe, even with fever or not. Conversely, influenza vaccinations are not contraindicated in the hospitalized population that got well after a short period of illness [62]. It also recommends the influenza vaccine should be contraindicated for children with severe allergic reactions, such as anaphylaxis, due to a previous dose or any component of any influenza vaccine (Table 2) [62].

**Table 2:** Summarization of the safety profile, contraindications, and precautions of three types of influenza vaccines

Vaccine Type	Adverse Events	Contraindications	Precautions
Egg-based inactivated influenza vaccine (egg-based IIV4s) [62]	Pain and other injection site reactions are frequently reported. They include symptoms such as pain, redness, and swelling at the injection site. Fever, malaise, myalgia, and other systemic symptoms can also occur after vaccination.	People who have experienced a severe or life-threatening allergic reaction (e.g., anaphylaxis) to any component of a specific influenza vaccine (other than egg protein).	Individuals who were hospitalized with an acute illness but are now well enough to be discharged can be vaccinated.
Cell culture-based inactivated influenza vaccine (ccIIV4) [62]	The safety profile of ccIIV4 is similar to that of other (egg-based) IIV4s.	ccIIV4 should not be administered to infants younger than 6 months of age.	History of a severe allergic reaction to a previous dose of any other influenza vaccine (i.e., any egg-based IIV, RIV, or LAIV of any valency).
Live attenuated influenza vaccine (LAIV4) [62]	Rhinitis (runny nose) and nasal congestion occur more commonly after LAIV. The most frequent side effects of LAIV include sore throat in adults, fever >100°F in children ages 2–6, and runny nose or nasal congestion in all age groups.	LAIV should not be administered to children aged <2 years of age or to adults aged ≥50 years of age. Furthermore, there are conditions and situations in which LAIV4 is contraindicated, such as when children or teenagers are taking aspirin or other salicylates (due to the link with Reye syndrome).	Asthma in children aged 5 years and older. Individuals who have other medical conditions that might place them at increased risk for complications.

Efficacy of different types of influenza vaccines in pediatrics

A study spanning nine influenza seasons revealed that the pooled VE for any influenza in pediatric populations was 46%, with the highest effectiveness observed in the six- to 59-month age group. It found that the effectiveness of influenza A (H3N2) virus infection was the lowest [13]. A review of evidence examines the effectiveness and efficacy of the influenza vaccination in young, healthy individuals under the age of 18. It was discovered that the efficacy of influenza vaccinations, with values ranging from 25.6% to 74.2%, and their effectiveness, with values ranging from 26% to 78.8%, are both statistically significant [63]. According to several studies, getting vaccinated against the flu dramatically lowers a child's risk of serious, life-threatening consequences. This risk includes a 75% reduction in severe, life-threatening influenza, a 41% reduction in hospitalizations, and a 50% reduction in ER visits caused by the flu [64]. Furthermore, the risk of flu-related death has been linked to a 51% reduction with flu vaccination and a 65% reduction in healthy children. Immunizations also reduced the risk of admission to the pediatric intensive care unit (PICU) due to influenza infection by 74% during flu seasons [64]. It also found that the risk of influenza illness in the overall population was reduced by 40% to 60% via the use of flu vaccination during well-matched seasons. It was found that the best effect of influenza vaccines is toward influenza A (H1N1) and influenza B viruses. However, the vaccines give low protection against influenza A (H3N2) viruses [64].

Attitudes and vaccination practices of parents with asthmatic children on the seasonal flu vaccine

Al-Qeremet et al. examined the knowledge, attitudes, and practices of parents of asthmatic children regarding influenza vaccine administration to their children [65]. Among patients who went to respiratory clinics at two hospitals in Jordan, there were 667 parents of children with asthma (62.8% of them were female) assigned to the study. The results showed that 60.4% of the children with asthma had never received a flu vaccination. According to 67% of individuals who had received the flu vaccination, there were not many side effects. Asthma duration was positively and significantly associated with increased vaccine hesitancy/rejection. The likelihood of vaccination hesitancy or rejection declines as attitudes toward flu vaccine scores rise. The top two excuses given for vaccine hesitancy/rejection were “I don't think my child needs it” (22.3%), followed by “I forget it” (19.5%) [65].

Guidelines from the American Academy of Pediatrics for preventing childhood influenza

Guidelines from the American Academy of Pediatrics recommend the use of influenza vaccination routinely for preventing childhood Influenza [66]. One of the most important recommendations of the AAP to prevent influenza infection is the use of influenza vaccines every year for all children whose age is six months or more and who have no contraindications to the vaccine. It recommends the use of vaccines for children at high risk to reduce hospitalization and death rates. Receiving influenza vaccines not only protects the children but also the surrounding community. It also reduces the severity of respiratory illnesses when they are co-circulated with the influenza virus. The AAP recommends the utilization of suitable influenza vaccines according to health status and age as soon as possible in the season. It has no recommendation for one vaccine over another. Another method for preventing the infection of the influenza virus is the use of antiviral chemoprophylaxis. It can be used as an adjuvant to the vaccines for certain conditions, such as children who have a risk of developing complications from influenza and who have not been immunized yet [19,66].

## Conclusions

In conclusion, receiving the influenza vaccine is generally immunogenic, effective, and safe in the pediatric population. The vaccinations elicit an immunological response that results in the formation of antibodies that defend against the influenza virus. Nonetheless, the review also emphasizes how crucial it is to take into account the child's age, health, and vaccination type while giving influenza shots. Certain influenza vaccines may be more suitable for specific age groups or individuals with underlying health conditions.

Furthermore, the review emphasizes the significance of ongoing monitoring and surveillance to ensure the efficacy and safety of influenza vaccines in children. Close monitoring of adverse events and regular assessment of vaccine efficacy contribute to continuous improvement in vaccine development and administration.
